# A Comparative Study of the Effect of Anxiety, Stress and Poor Sleep on Multipara and Nullipara Pregnant Women

**DOI:** 10.7759/cureus.86205

**Published:** 2025-06-17

**Authors:** Pragati Divedi, Deepti Dwivedi, Nimarpreet Kaur, Pragyashaa Chaudhary, Sunil Chamola

**Affiliations:** 1 Department of Obstetrics and Gynecology, Uttar Pradesh University of Medical Sciences, Saifai, IND; 2 Department of Physiology, Faculty of Medicine and Health Sciences, Shree Guru Gobind Singh Tricentenary (SGT) University, Gurugram, IND; 3 Department of Community Medicine, Maharishi Markandeshwar College of Medical Sciences and Research, Ambala, IND

**Keywords:** antenatal poor sleep, anxiety, gestational stress, perceived stress, sleep health

## Abstract

Introduction: Sleep is an essential component of well-being. It is defined as a reversible behavioral state of perception disengagement from unresponsiveness to the environment. Sleep deprivation leads to stress, leading to mental issues like mood disorders and depression. This study has a prime focus on how the psychological parameters, like anxiety and stress, are affected by sleep deprivation during pregnancy and differentiates these in nullipara and multipara women.

Aim and objectives: The aim of this study was to study the effect of sleep health on the psychological health of pregnant women. The objectives were (i) to assess the quality of sleep in pregnant women by using the Pittsburgh Sleep Quality Index (PSQI 21 items), (ii) to compare the quality of sleep in multipara and nullipara pregnant women by using the Pittsburgh Sleep Quality Index (PSQI), (iii) to compare the psychological health in multipara and nullipara pregnant women by using the Perceived Stress Scale (PSS-10) & Generalized Anxiety Disorder-7 (GAD-7), and (iv) to compare the strength of association between sleep health and psychological health in multipara and nullipara pregnant women.

Materials and methods: This is a cross-sectional study, done on 346 naturally conceived singleton literate pregnant women of age 18-41 years at eight weeks to 28 weeks of pregnancy who visited FMHS SGT Hospital Obstetrics & Gynecology Department following our inclusion criteria and gave consent during the study period. The study was conducted for a duration of six months from July 2024 to December 24.

Results: The study results show that out of 346 women, 200 (57.80%) were nulliparous and 146 (42.2%) were multiparous. Among 346 women, 186 (53.76%) had poor sleep with a mean ±SD of 6.28 *±* 2.56, out of which 73 (39.24%) were multipara and 113 (60.75%) pregnancies were nullipara. A positive correlation of 0.53 was present between PSS-10 and PSQI global score. Also, a strong correlation of r=0.57 was present between the PSQI global score and GAD-7.

Conclusion: Increased levels of stress and anxiety had a vicious direct effect related to the poor quality of sleep by acting over HPA axis activity. Poor sleep health is an important indicator of the mental health of women during pregnancy. Multiparous women have better coping strategies than nulliparous women as they are more experienced from their previous pregnancies.

## Introduction

Sleep is a key component of health. It has an essential role in various reparative processes of the body, along with the removal of toxins, release of hormones, and memory consolidation [1,2]. Hormonal changes affect the psychological health of women including impaired sleep quality in women. During pregnancy, the hypothalamo-pituitary-adrenal (HPA) axis has reduced stress responsiveness along with the anti-inflammatory profile of the immune system [3]. Maternal stress stimulates the downregulated HPA axis and inflammatory responses during pregnancy, resulting in maternal illness during pregnancy. This increases behavioral changes and disordered sleep [4].

Sleep insufficiency has been linked to the leptin hormone of adipose tissue, responsible for appetite suppression and increased concentration of ghrelin hormone, which stimulates the appetite. Adults with chronic sleep loss report excess mental distress, depressive symptoms, anxiety, and alcohol use [5]. 

Pregnancy is different for the same woman, and the consequences of various physiological changes even vary within each trimester of the same pregnancy, which has a huge impact on the sleep quality and psychological health of pregnant women.

This study was planned to provide valuable insights into the influence of sleep quality on the psychological state during pregnancy. This study has a primary focus on how the psychological parameters like anxiety and stress are affected by sleep health during pregnancy and differentiates these in nullipara and multipara pregnancies. The mental health of women during pregnancy has been discussed for a long time, but how it is different in women with multipara and nullipara pregnancies affected by sleep deprivation is analyzed in this study.

## Materials and methods

This cross-sectional study was done on 386 naturally conceived singleton literate pregnant women at eight weeks to 28 weeks of pregnancy who visited FMHS SGT Hospital Obstetrics & Gynecology Department and gave consent during the study period. Naturally conceived singleton literate pregnant women at 8-28 weeks of pregnancy of age 18-41 years participated in this study. The duration of the study was six months, i.e., July 2024 to December 2024. Forty pregnant women were excluded. They were those with any history of medical and psychiatric illnesses (medical and psychological), history of pregnancy loss, drugs, alcohol, and hormonal therapy, and unpredictable conditions such as travelling to different time zones and night shift workers.

**Figure 1:**
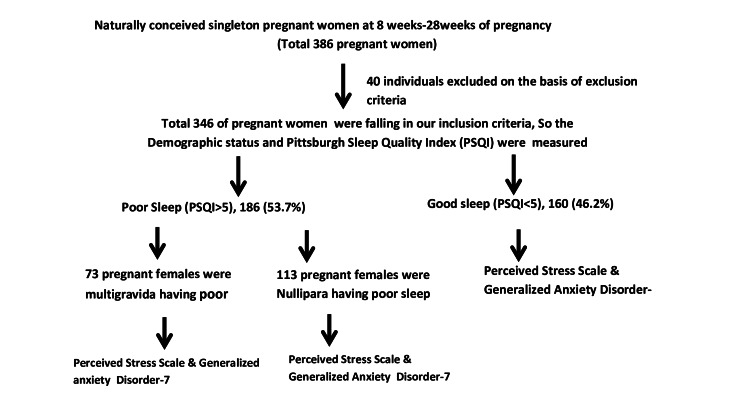
Study design

The study was done by using widely used and validated tools shown in Figure 1.

The study tool was divided into four different sections: (i) Demographic status (eight items), (ii) Perceived stress score (10 items) [6], (iii) Pittsburgh Sleep Quality Index (PSQI 21 items) [7], and (iv) Generalized Anxiety Disorder (seven items) [8]. Descriptive statistics and Pearson correlation test were applied using IBM SPSS Statistics for Windows, Version 26 (Released 2019; IBM Corp., Armonk, New York, United States).

Pittsburgh Sleep Quality Index (PSQI)

The sleep quality was measured by using this scale system. It is a free validated questionnaire categorized into seven components: sleep quality, latency, duration, efficiency, sleep disturbances, use of medications, and daytime dysfunction. It contains 21 questions. The global score of PSQI ranges between 0 and 21. A score less than 5 indicates good sleep quality, ‘0’ indicates no difficulty, and a total score greater than 5 indicates poor sleep quality. The scale has been widely used in various studies with high reliability and validity and the Cronbach’s alpha of this index is 0.811.

Perceived Stress Scale (PSS)

This is the most widely used scale to measure perceived stress. Each question is scored from 0 (never) to 4 (very often), with a total possible score range of 0 to 40. The higher score interprets a higher degree of stress: Scores ranging from 0 to 13 are considered as low stress; scores ranging from 14 to 26 are considered as moderate stress; scores ranging from 27 to 40 are considered as high perceived stress

Generalized Anxiety Disorder-7 (GAD-7)

The GAD-7 is a seven-item self-report scale developed to assess the defining symptoms of anxiety. The various items are graded on a 4-point Likert-type scale (0=not at all to 3=nearly every day). Scores range from 0 to 21, with higher scores indicating more severe GAD symptoms.

## Results

The study results show that out of 346 women, 200 were nulliparous, and 146 (42.2%) were multiparous. Among 346 women, 186 (53.76%) had poor sleep with a mean ± SD of 6.28 ± 2.56, out of which 73 were multipara and 113 pregnancies were nullipara.

Table 1 shows that the majority of the population falls in the >30 years age group, comprising 208 individuals, while a smaller portion is in the 25-30 years age group, making up 138. Nulliparous represents the majority, with 200 women, while multiparous makes up a smaller group of 146 women. The majority of women were married (344 women), while a very small proportion (two women) were single. Most participants were in the second trimester, accounting for 214 women. The first-trimester group had 80 pregnant women, while the third-trimester group had 52 pregnant women. The majority of the sample belongs to the middle class, comprising 211 women. Lower-class women were 93, while a smaller group was in the upper class (42 women).

**Table 1:** Demographic characteristics of pregnant women PSQI: Pittsburgh Sleep Quality Index

Parameters		N (%)
Age	25-30 years	138
	>30 years	208
Global PSQI score (>5)	<30 years	117
	>30 years	69
Global PSQI score (<5)	<30 years	21
	>30 years	139
Parity	Nulliparous	200
	Multiparous	146
Marital status	Married	344
	Single	2
Gestational age	1^st^ Trimester	80
	2^nd^ Trimester	214
	3^rd^ Trimester	52
Socioeconomic status	Lower class	93
	Middle class	211
	Upper class	42

**Figure 2:**
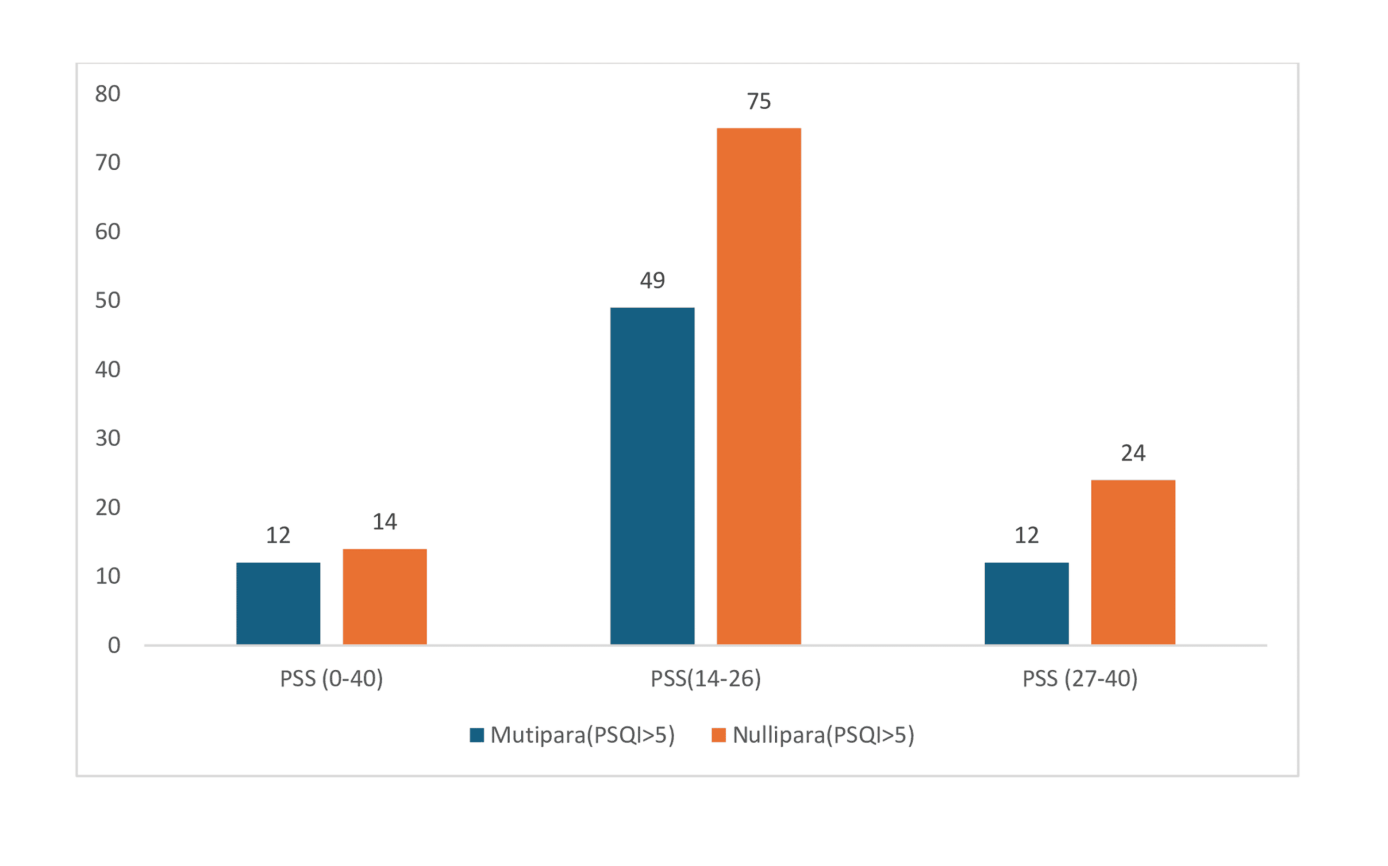
Comparison of the perceived stress level and PSQI score of multipara and nullipara women with poor sleep quality PSQI: Pittsburgh Sleep Quality Index; PSS: Perceived Stress Scale

Table 2 and Figure 2 show that pregnant women with poor sleep quality were 186 while 160 women had good sleep quality. A small proportion of women with poor sleep quality reported mild stress. This is observed in both multipara (12; 16.44%) and nullipara (14; 12.38%) women, while a significantly higher proportion of women with good sleep quality experienced mild stress, particularly nullipara women (43; 58.10%), followed by multipara women (33; 38.37%).

**Table 2:** Comparison of sleep quality and stress between multipara and nullipara women by using the PSQI global score and PSS-10 PSQI: Pittsburgh Sleep Quality Index; PSS: Perceived Stress Scale

Perceived stress score (0-40)	Poor sleep quality (PSQI score>5) 186 (53.75%)		Good sleep quality (PSQI score<5) 160 (46.25%)	
	Multipara women (73; 39.24%)	Nullipara women (113; 60.75%)	Multipara women (86; 53.75%)	Nullipara women (74; 46.25%)
Mild stress (Score 0-13)	12 (16.44%)	14 (12.38%)	33 (38.37%)	43 (58.10%)
Moderate stress (Score 14-26)	49 (67.12%)	75 (66.38%)	29 (33.72%)	23 (31.08%)
Severe stress (Score 27-40)	12 16.44%)	24 (21.24%)	24 (27.90%)	8 (10.81%)

A significant majority of women with poor sleep quality experienced moderate stress. Among multipara women, 49 (67.12%) reported moderate stress, while 75 (66.38%) nullipara women also reported moderate stress. Moderate stress is less common among those with good sleep quality compared to poor sleep quality. Only 29 (33.72%) multipara women and 23 (31.08%) nullipara women with good sleep quality reported moderate stress. A smaller proportion of women with poor sleep quality experienced severe stress. Twelve (16.44%) multipara women and 24 (21.24%) nullipara women with poor sleep quality reported severe stress. A higher proportion of multipara women with good sleep quality experienced severe stress 24 (27.90%) compared to nullipara women with good sleep quality 8(10.81%). The chi-square value is 75.32 with a p-value less than 0.001, which explains that there is an association between sleep pattern and stress.

Table 3 shows that among women with poor sleep quality, minimal anxiety (Score 0-4) was reported by a small percentage (five (2.69%) multipara women and 15 (8.06%) nullipara women). Mild anxiety (score 5-9) was reported by 13 (6.98%) multipara women and eight (4.30%) nullipara women. Moderate anxiety (score 10-14) was significantly higher among women with poor sleep quality, with 38 (20.43%) multipara women and 68 (36.56%) nullipara women reporting moderate anxiety. Severe anxiety (Score >15) was reported by 17 (9.14%) multipara women and 22 (11.83%) nullipara women. Among women with good sleep quality, minimal anxiety (Score 0-4) was reported by 13 (8.13%) multipara women and six (3.75%) nullipara women, mild anxiety (Score 5-9) was reported by 29 (18.12%) multipara women and 23 (14.37%) nullipara women, moderate anxiety (Score 10-14) was reported by 24 (15.00%) multipara women and 36 (22.50%) nullipara women, and severe anxiety (Score >15) was reported by 20 (12.5%) multipara women and nine (5.63%) nullipara women.

**Table 3:** Comparison of sleep quality and anxiety between multipara and nullipara women by using the PSQI global score and PSS-10 PSQI: Pittsburgh Sleep Quality Index; PSS: Perceived Stress Scale

Generalized anxiety disorder 7 (0-21)	Poor sleep quality (PSQI score>5) (186 women)		Good sleep quality (PSQI score<5) (160 women)	
	Multipara women (73)	Nullipara women (113)	Multipara women (86)	Nullipara women (74)
Minimal anxiety (Score 0- 4)	5	15	13	6
Mild anxiety (Score 5-9)	13	8	29	23
Moderate anxiety (Score 10-14)	38	68	24	36
Severe anxiety (Score >15)	17	22	20	9

Table 4 shows that the Mean±SD perceived stress score for multipara women with poor sleep quality is 10.73 ± 3.65, while in nullipara women, the Mean±SD perceived stress score with poor sleep quality was 21.03 ± 4.90. This indicates a statistically significant difference between the two groups. The Mean±SD generalized anxiety disorder scale score for multipara women with poor sleep quality is 7.87 ± 3.21, and the Mean±SD generalized anxiety disorder scale score for nullipara women with poor sleep quality is 12.31 ± 1.36. The p-value is <0.0001, which shows a highly statistically significant difference between the two groups.

**Table 4:** Psychological assessment of poor quality sleep PSQI: Pittsburgh Sleep Quality Index; PSS: Perceived Stress Scale; GAD: Generalized Anxiety Disorder

Psychological assessment scale	Poor quality sleep		t-value	p-value
	Multipara N1=73 (39.24%)	Nullipara N2=113 (60.75%)		
Perceived stress scale score	10.73±3.65	21.03±4.90	15.40	<0.0001
Generalized Anxiety Disorder scale Score	7.87± 3.21	12.31±1.36	13.02	<0.0001
PSQI and PSS r-value	0.53			
PSQI and GAD r-value	0.57			

Table 5 shows that out of 80 pregnant women in the first trimester, 58 (23.13%) had poor sleep quality among which 31 (53.45%) were in moderate to high level of stress (PSS≥14), and 36 (62.07%) had moderate to high anxiety with (GAD-7 ≥ 10). Similarly, out of 214 pregnant women in the second trimester, 102 (47.67%) had poor sleep quality among which 31 (53.45%) were in moderate to high level of stress (PSS≥14), and 49 (48.04%) had moderate to high anxiety with (GAD-7≥ 10). Among 52 pregnant women in the third trimester, 34 (65.38%) had poor sleep quality, among which 21 (61.76%) were in a moderate to high level of stress (PSS≥14), and 24 (70.59%) had moderate to high anxiety with (GAD-7≥ 10).

**Table 5:** Comparison of sleep health and psychological health in multipara and nullipara pregnant women PSQI: Pittsburgh Sleep Quality Index; PSS: Perceived Stress Scale; GAD: Generalized Anxiety Disorder

	First trimester (80 women; 23.13%)		Second trimester (214 women; 61.84%)		Third trimester (52 women; 15.03%)	
	Poor sleep quality (PSQI>5) 58	Good sleep quality (PSQI<5) 22	Poor sleep quality (PSQI>5) 102	Good sleep quality (PSQI<5) 112	Poor sleep quality (PSQI>5) 34	Good sleep quality (PSQI<5) 20
Stress (PSS ≥ 14)	31	5	49	27	21	8
Anxiety (GAD-7 ≥10)	36	9	53	32	24	9

## Discussion

The study conducted by Kiper et al. showed that sleep quality is better in younger pregnant women. This finding differed from the present study, where 117(62.98%) women aged less than 30 years had poor quality sleep [9]. The 214 (60.98%) women were from the middle socioeconomic status, out of which 98 (45.80%) had poor sleep. The previous conducted study explains our results by the fact that, as previously suggested in the literature, normally, education and socioeconomic status are likely to result in good mental health [10-12]. Our study results show that women during pregnancy have poor quality of sleep. The percentage of poor quality sleep was further more in nulliparous (186; 53.76%) women than in multiparous women (73; 39.24%). This finding is in concordance with the study conducted in Taiwan & Iran [13,14]. Although a Vietnamese study conducted on 119 healthy pregnancies reported contradictory results [15].

The findings of the present study suggested that there was a statistically highly significant difference in stress level between multipara (10.73±3.65) and nullipara women (21.03±4.90). The possible reason could be that multipara women have better coping strategies as they have previous pregnancy experience. Further, the stress levels of the moderate to severe category with poor sleep quality were positively associated with perceived stress score, and a correlation of 0.53 was present between them. This finding is concordant with the previously done study on the Chinese population by Gao [16].

The FinnBrain birth cohort study conducted on 78 healthy pregnant women shows deterioration in sleep quality across the pregnancy, along with depressive & anxiety-related symptoms. These results were similar to the present study, where out of 186 poor sleep quality, 145 (77.96%) had moderate to severe levels of generalized anxiety score. Among these, 55 (29.57%) were multipara and 90 (48.39%) were nullipara [17,18].

Further, we can elaborate on the linkage between anxiety and obstetric history on the basis of our results, as the generalized anxiety disorder score was statistically highly significant for multipara (7.87±3.21) and nullipara (12.31±1.36). The possible reason for this could be that nullipara women have less fear of the outcome of pregnancy in comparison to multipara women in the Indian population. These results correspond with the findings of a study done on pregnant women in the Netherlands who reported more episodes of anxiety symptoms in multiparity, with severe nausea and extreme fatigue [19,20].

The study conducted on 148 pregnant women determined that the stress level of pregnant women was high in all three trimesters, and the worst sleep quality and stress were reported in the third trimester. These findings were in accordance with the present study finding where among the poor sleep quality pregnant women, 31 (53.45%) women in the first trimester, 49 (48.04%) women in the second trimester and 24 (70.59%) women in the third trimester were suffering from moderate to high level of stress and stress worsened in third-trimester women. Thus the sleep quality decreases as the pregnancy stress increases and worsens in the third trimester of pregnancy [21,22].

The pattern of anxiety was also following a similar trend, as in the first trimester, 36(62.07%), second trimester, 53(51.96%), and in the third trimester, 24 (70.59%) pregnant women had poor sleep quality. The anxiety score increases in women in their third trimester, with the deterioration in sleep quality. Further, various previous research studies have documented that maternal anxiety sleep quality, and life stress have a strong association [23,24]. However, this is in contrast to the study published with the 630 pregnant women from Spain by Pascal et al. They had different results stating that anxiety and depressive symptoms do not have a homogeneous trend. They found a low correlation between sleep quality and stress and anxiety [25].

The authors recognize that there were a few areas, like a single-center study, and single point of contact, and a short duration of study were major limitations. Due to the uniformity of the rural population, the analysis was not powered to control for multiple potential confounders. However, we did not control for age, but the maximum responders were in the younger age group. 

## Conclusions

Increased levels of stress and anxiety had a vicious direct effect related to the poor quality of sleep by acting on HPA axis activity. Poor sleep health is an important indicator of the mental health of women during pregnancy. Multiparous women have better coping strategies than nulliparous women as they have more experience from their previous pregnancies. The future scope of study includes observing the relationship between pregnancy outcome and sleep and psychological health. The psychological screening and sleep health assessment during pregnancy should be an essential component of the antenatal checkups. The antenatal visits of pregnant women with the assessment of psychological health and sleep health could be a very important method to prevent the adverse effects of pregnancy outcomes induced by them. Sleep health education and maintenance of good sleep hygiene will have a positive impact on psychological health during pregnancy. The outcome of this study envisages attention to sleep health and psychological health during pregnancy.
